# Targeting mitochondria as a potential therapeutic strategy against radioresistance in cancer

**DOI:** 10.3389/fonc.2026.1849057

**Published:** 2026-06-05

**Authors:** Nazia Nazam, Shaikh Sadiya, Saba Khan, Aroonima Misra, Niti Sureka, Kirti Panwar, Neelam Sahani, Sufian Zaheer

**Affiliations:** 1 Pediatric Surgery, School of Medicine, University of Alabama at Birmingham, Birmingham, AL, United States; 2 Department of Pathology, Vardhman Mahavir Medical College and Safdarjung Hospital, New Delhi, India; 3 Department of Biochemistry, Integral Institute of Medical Sciences &Research, Lucknow, Uttar Pradesh, India; 4 Advances Molecular Diagnostics and Research Facility, Indian Council of Medical Research (ICMR)-National Institute of Child Health and Development Research, New Delhi, India; 5 Department of Pathology, Holy Family Hospital, New Delhi, India

**Keywords:** cancer, mitochondria, radioresistance, radiosensitization, radiotherapy

## Abstract

Radioresistance remains a major barrier to effective cancer therapy, contributing to tumor persistence, recurrence, and poor clinical outcomes. Increasing evidence identifies mitochondria as central regulators of radiation response through their multifaceted roles in cellular bioenergetics, redox homeostasis, mitochondrial DNA (mtDNA) maintenance, apoptotic signaling, and mitochondrial dynamics. Radioresistant tumor cells undergo profound metabolic reprogramming characterized by enhanced oxidative phosphorylation (OXPHOS), glycolytic plasticity, glutaminolysis, and pentose phosphate pathway activation, enabling sustained ATP generation, antioxidant defense, and efficient DNA repair under radiation stress. In parallel, mitochondrial reactive oxygen species (ROS) signaling is tightly modulated by antioxidant systems including glutathione, superoxide dismutase, catalase, and NRF2-driven pathways, thereby limiting radiation-induced oxidative injury. Alterations in mitochondrial fusion and fission dynamics, particularly Drp1-mediated fission, further support tumor survival by promoting mitophagy, metabolic adaptation, and resistance to apoptosis. Additionally, enhanced mtDNA repair and mitochondrial biogenesis preserve mitochondrial integrity in irradiated cancer cells. Dysregulation of mitochondria-mediated intrinsic apoptotic pathways, including aberrant expression of Bcl-2 family proteins, further facilitates evasion of radiation-induced cell death. This review comprehensively examines the molecular mechanisms by which mitochondria contribute to tumor radioresistance and critically discusses emerging mitochondria-targeted therapeutic strategies aimed at improving radiosensitivity. These include OXPHOS inhibitors, glycolytic and glutaminase inhibitors, ROS-modulating agents, mitochondrial dynamics regulators, nanoparticle-based mitochondrial targeting systems, and combinatorial approaches integrating radiotherapy with immunotherapy or DNA damage response inhibitors. By integrating mechanistic insights with emerging preclinical and clinical evidence, this review highlights mitochondria as actionable therapeutic vulnerabilities and underscores the translational potential of mitochondrial-targeted radiosensitization strategies for improving outcomes in resistant malignancies.

## 1 Introduction

**Figure 1:**
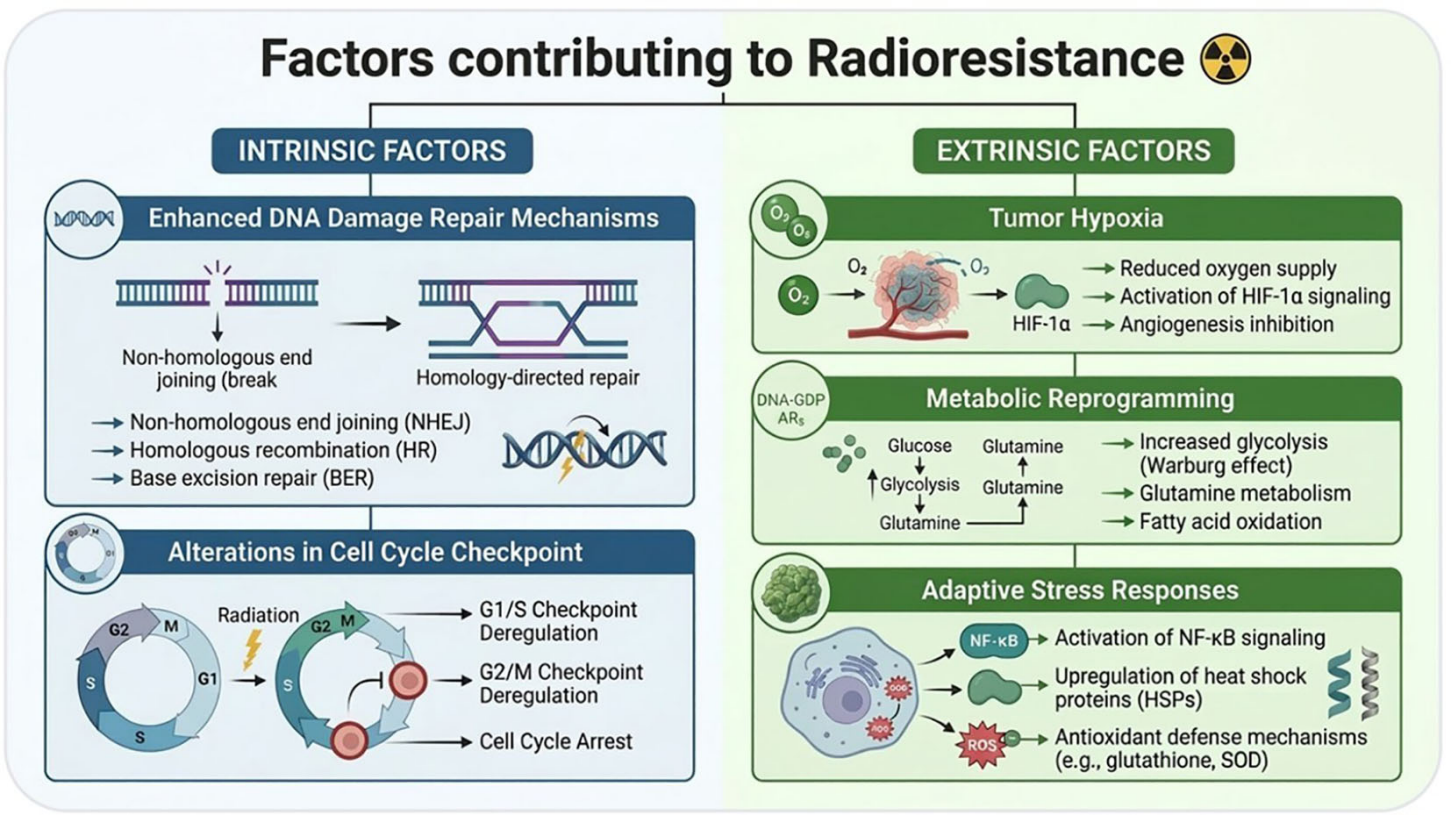
Intrinsic and extrinsic determinants of tumor radioresistance. Radioresistance arises from the interplay of cell-autonomous mechanisms (Intrinsic) and microenvironmental adaptations (extrinsic) that collectively attenuate radiation-induced cytotoxicity. Intrinsic factors include: (i) enhanced DNA damage repair via NHEJ, HR, and BER, restoring genomic integrity; and (ii) aberrant cell cycle checkpoint regulation that prolongs arrest, enabling repair and evasion of mitotic catastrophe. Extrinsic factors encompass: (i) tumor hypoxia, which attenuates radiation-induced ROS while activating HIF-1α and pro-survival signaling; (ii) metabolic reprogramming (aerobic glycolysis, glutaminolysis, fatty acid oxidation) that sustains bioenergetics and buffers oxidative stress; and (iii) adaptive stress responses (NF-κB activation, HSP induction, antioxidant defenses) that scavenge residual ROS and suppress apoptosis. Together, these pathways diminish radiotherapy efficacy and confer a robust radioresistant phenotype.

Radiotherapy remains a cornerstone of cancer treatment, employed across a broad spectrum of malignancies either as a definitive therapeutic modality or in combination with surgery, chemotherapy, targeted therapy, and immunotherapy (1, 2). Despite significant technological and biological advances in radiation oncology, the therapeutic efficacy of radiotherapy is frequently compromised by the emergence of radioresistance, a multifactorial phenomenon that promotes tumor persistence, recurrence, metastasis, and poor clinical outcomes (3, 4). Radioresistance develops through a complex interplay of intrinsic tumor cell adaptations and extrinsic microenvironmental influences that collectively attenuate radiation-induced cytotoxicity (5–7). Intrinsic mechanisms include enhanced DNA damage repair pathways such as non-homologous end joining (NHEJ), homologous recombination (HR), and base excision repair (BER), which facilitate efficient repair of radiation-induced DNA lesions and preserve tumor cell survival (8, 9). Dysregulation of cell cycle checkpoint signaling, particularly through ATM/ATR-mediated pathways, further provides tumor cells with extended time for DNA repair and evasion of mitotic catastrophe (10, 11). Extrinsic mechanisms, including tumor hypoxia, metabolic reprogramming, and adaptive stress responses, also contribute substantially to resistance. Hypoxic tumor regions limit radiation-induced reactive oxygen species (ROS) formation while activating hypoxia-inducible factor-1α (HIF-1α)-driven survival pathways (6, 12, 13). Simultaneously, metabolic adaptations such as enhanced glycolysis, glutaminolysis, and fatty acid oxidation sustain bioenergetic demands, maintain redox balance, and support DNA repair under radiation stress (14, 15). In addition, activation of antioxidant defenses, heat shock proteins, and pro-survival signaling pathways including NF-κB enables tumor cells to neutralize oxidative damage and evade apoptosis (16, 17). Together, these mechanisms create a highly adaptive and treatment-resistant tumor phenotype, highlighting the urgent need for novel radiosensitizing strategies (**Figure 1**; **Table 1**).

**Table 1:** Summary of mechanisms behind cancer cell resistance to radiotherapy.

	Mechanism	Key players/mediators	Function/role
1	Dysregulated Cell Cycle	Chk1, Chk2, p53, p21, ATM, ATR	Disrupts cell cycle checkpoints, allowing cells to evade radiation-induced cell death.
	Enhanced DNA Damage Repair	MDC1, PARPs, ATM, BRCA1, DNA-PKcs, rH2AX, WEE1, 53BP1, NBS1/hMRE11/hRAD50, Ku70/Ku80 (upregulate DNA damage response (DDR))	Repairs radiation-induced DNA damage to promote cell survival.
3	Activation of Survival Signaling Pathways	PI3K/AKT, MAPK, and NF-κB	Promote cell survival and radioresistance by preventing apoptosis and enhancing repair mechanisms.
4	Evasion of Apoptosis	Overexpression of anti-apoptotic proteins (e.g., Bcl-2, Bcl-xL) and loss of pro-apoptotic factors (e.g., p53, Bax), IAPs	Suppresses apoptotic pathways to enhance cell survival.
5	Epigenetic Modifications	DNA methylation, histone modifications, and miRNA dysregulation	alter gene expression patterns that influence radiosensitivity.
4	Tumor Microenvironment		
	- Hypoxia	Low oxygen levels	Reduces radiation efficacy by limiting oxygen-dependent DNA damage.
	- Inflammation	Chronic inflammatory signals	Promotes tumor survival and resistance.
	- Immunosuppression	Immune evasion mechanisms	Prevents immune-mediated destruction of cancer cells.
	- CAFs	Fibroblast-derived signals	Supports tumor growth and resistance to therapy.
5	Cancer Stem Cells (CSCs)		
	- Immunosuppression	Immunosuppressive signals	Inhibits anti-tumor immune responses.
	- Survival Mechanisms	Autophagy, apoptosis resistance, DNA repair	Enhances survival under stress conditions.
	- Low ROS Levels	Reduced reactive oxygen species	Minimizes radiation-induced oxidative damage.
	- Epithelial–Mesenchymal Transition (EMT)	High cellular plasticity	Increases resistance and metastatic potential.
	- Quiescence	Dormant state	Reduces susceptibility to radiation.
	- Signaling Pathways	Wnt, Notch, Hedgehog, TGF-ß, PI3K/AKT/mTOR	Promotes survival and resistance.
6	Metabolic Reprogramming		
	- Glucose Metabolism	Altered glycolysis	Fuels cancer cell survival.
	- Lipid Metabolism	Changes in lipid processing	Enhances resistance.
	- Glutamine/Serine Metabolism	High expression of glutamine synthetase, purine, and serine protease inhibitor E2	Supports tumor growth.
7	Exosomes and EVs		
	- Macrophage Polarization	M1 to M2 tumor-associated macrophage shift	Promotes a pro-tumor microenvironment.
	- Non-Coding RNAs (ncRNAs)	ncRNA-mediated signaling	Regulates resistance mechanisms.
8	Ferroptosis Resistance	GPX4, CoQ-FSP1, iron metabolism, PUFA-PL	Prevents iron-dependent cell death (ferroptosis), enhancing survival.
9	Autophagy-Induced Resistance	Radiation-induced autophagy	Promote survival by degrading damaged organelles and proteins, reducing cell death.

γH2AX, Phosphorylated H2AX; 53BP1, p53-Binding Protein 1; ATM, Ataxia-telangiectasia mutated; ATR, ATM and Rad3-related; BRCA1, Breast Cancer Type 1 Susceptibility Protein; CAF, Cancer associated Fibroblast; DNA-PKcs, DNA-dependent protein kinase catalytic subunit; GPX4, Glutathione peroxidase 4; IAPs, Inhibitor of apoptosis proteins; MDC1, Mediator of DNA Damage Checkpoint; PARP, Poly(ADP-ribose) polymerase; PARPs, Poly [ADP-ribose] Polymerases; PUFA-PL, Polyunsaturated fatty acid-containing phospholipid; ROS, Reactive oxygen species; PUFA-PL, Polyunsaturated fatty acid-containing phospholipid; TAM, Tumor-associated macrophages.

Among the numerous cellular determinants of radioresistance, mitochondria have emerged as critical regulators of radiation response (18–20). Beyond their canonical role in oxidative phosphorylation (OXPHOS) and ATP synthesis, mitochondria function as central hubs for ROS generation, redox signaling, metabolic plasticity, apoptosis regulation, and mitochondrial DNA (mtDNA) maintenance (21, 22). Ionizing radiation exerts cytotoxic effects largely through ROS-mediated oxidative stress, which induces DNA strand breaks, lipid peroxidation, protein oxidation, and mitochondrial dysfunction (23–25). However, radioresistant cancer cells frequently adapt to these insults by enhancing mitochondrial antioxidant defenses, rewiring mitochondrial metabolism, and modulating mitochondrial dynamics to preserve survival under genotoxic stress (26, 27). Increased mitochondrial plasticity allows tumor cells to switch between OXPHOS and glycolysis according to metabolic demands, thereby sustaining ATP production while minimizing oxidative injury (28, 29). Concurrently, mitochondrial fusion-fission dynamics and mitophagy play essential roles in maintaining mitochondrial quality control, removing damaged organelles, and promoting cellular adaptation to radiation-induced stress (30, 31). Furthermore, efficient mtDNA repair mechanisms and mitochondrial biogenesis help preserve mitochondrial integrity and bioenergetic function in irradiated cancer cells, further reinforcing radioresistance (32, 33). Dysregulation of mitochondria-mediated apoptotic pathways, particularly through altered expression of Bcl-2 family proteins, also contributes significantly to evasion of radiation-induced cell death.

Given the central role of mitochondria in coordinating metabolic adaptation, oxidative stress regulation, DNA repair, and apoptotic resistance, targeting mitochondrial pathways has emerged as a promising therapeutic strategy for overcoming radioresistance (34–36). Pharmacological approaches aimed at disrupting mitochondrial metabolism, enhancing ROS accumulation, inhibiting mitochondrial antioxidant defenses, modulating mitochondrial dynamics, impairing mtDNA repair, and restoring mitochondrial-mediated apoptosis have demonstrated encouraging radiosensitizing effects in multiple preclinical and clinical studies (34–36). In particular, inhibitors of OXPHOS, glycolysis, glutaminase activity, and mitochondrial respiratory complexes, as well as ROS-modulating agents and mitochondria-targeted nanoparticles, are increasingly being explored as adjuncts to radiotherapy (37). Additionally, combinatorial strategies integrating mitochondrial-targeted therapies with immunotherapy and DNA damage response inhibitors are expanding the therapeutic landscape for resistant malignancies (34–36).

In this review, we provide a comprehensive and mechanistically focused overview of the role of mitochondria in tumor radioresistance. We discuss the molecular pathways through which mitochondrial metabolism, ROS regulation, mitochondrial dynamics, mtDNA repair, and apoptotic signaling contribute to resistance against radiotherapy. Furthermore, we critically evaluate emerging mitochondrial-targeted therapeutic strategies designed to enhance radiosensitivity and improve treatment outcomes in resistant cancers. By integrating current mechanistic insights with evolving translational evidence, this review highlights mitochondria as actionable therapeutic vulnerabilities and underscores their potential as central targets in the development of next-generation radiosensitizing therapies.

## 2 Mitochondrial role in radioresistance

### 2.1 Mitochondrial metabolism and energy production in radioresistant cancer cells

Mitochondria are central to cellular metabolism, and radioresistant cancer cells undergo metabolic reprogramming to meet energy demands and counteract radiation−induced stress (38). These cells exhibit metabolic plasticity, switching between OXPHOS and glycolysis to sustain ATP supply while minimizing oxidative damage (20, 26, 38). Enhanced mitochondrial respiration, including upregulation of electron transport chain (ETC) components such as complex I, supports bioenergetic demands and maintains mitochondrial membrane potential during radiotherapy (26, 39). Complex I defects can also promote radioresistance by activating DNA damage repair pathways (40). Conversely, mitochondrial complex I inhibitors increase lactate release (indicating metabolic flux shift) and sensitize tumor cells to radiation (41). Upregulated glycolysis, marked by increased glucose uptake and lactate production, supports proliferation, DNA repair, and redox balance, further contributing to radioresistance (42). Disrupting mitochondrial function augments radiosensitivity in prostate cancer cells (43), and metabolic reprogramming is implicated in rectal cancer radioresistance, with altered metabolites as potential predictive markers (44). In colorectal cancer, mitochondrial dysfunction induces radioresistance via specific signaling pathways (40). Thus, metabolic reprogramming particularly enhanced glycolysis and mitochondrial dysfunction, emerges as a conserved hallmark of radioresistance across diverse tumor types, highlighting the potential of targeting these pathways for therapeutic benefit.

Beyond ATP production, mitochondria regulate metabolic signaling that influences radioresistance. Upregulation of tricarboxylic acid (TCA) cycle intermediates provides metabolites for anabolic processes and redox balance (39, 45, 46). For instance, α−ketoglutarate (α−KG) mediates epigenetic modifications that enhance stem−like properties and resilience to radiation (46–48). Mutations in isocitrate dehydrogenase 1 (IDH1) lead to D−2−hydroxyglutarate (D−2HG) accumulation, which inhibits α−KG−dependent dioxygenases, causing epigenetic changes, DNA hypermethylation, and impaired differentiation (49, 50).

Metabolic cross−talk between mitochondria and the pentose phosphate pathway (PPP) enhances nucleotide biosynthesis and antioxidant capacity, enabling efficient DNA repair after radiation−induced damage (51, 52). The PPP supplies ribose−5−phosphate for nucleotide synthesis and NADPH for redox homeostasis, counteracting radiation−induced ROS accumulation (51). Targeting these interconnected pathways is a promising radiosensitization strategy. Dual inhibition of glucose−6−phosphate dehydrogenase (G6PD) and transketolase (using 6−aminonicotinamide and oxythiamine) reduced thyroid cancer cell viability by increasing ROS, endoplasmic reticulum stress, and apoptosis (53). Combining the glycolysis inhibitor 2−deoxy−D−glucose (2−DG) with the mitochondrial complex I inhibitor metformin enhanced radiosensitivity in neuroblastoma cells by disrupting ATP production (54). Thus, mitochondrial metabolic reprogramming supports cancer cell radioresistance through multiple energy pathways and altered signaling, presenting potential therapeutic targets to improve radiotherapy efficacy.

### 2.2 Mitochondrial ROS and oxidative stress regulation in radiation response

ROS mediate the cytotoxic effects of radiotherapy by inducing oxidative damage to DNA, proteins, and lipids (55, 56). Ionizing radiation generates ROS directly *via* water radiolysis (producing hydroxyl radicals and H_2_O_2_) and indirectly by damaging mtDNA and ETC components, which increases mitochondrial ROS production (57). Impaired electron transport leads to superoxide anion formation, propagating DNA double−strand breaks (DSBs), protein oxidation, and lipid peroxidation, ultimately triggering apoptosis, necrosis, or senescence (58). However, cancer cells with high mitochondrial plasticity and adaptive antioxidant responses manage oxidative stress and evade death, promoting radioresistance (20).

Radioresistant tumors mitigate ROS damage through several mechanisms. Antioxidant enzymes (superoxide dismutase-SOD, catalase, glutathione peroxidase, peroxiredoxins) are upregulated to neutralize ROS and maintain redox homeostasis (59–61). In a radioresistant glioblastoma clone, radiation exposure induced up to a fivefold increase in these enzymes (62). Elevated glutathione (GSH) levels further scavenge ROS and support glutathione peroxidase activity (63, 64). The transcription factor NRF2 drives expression of detoxifying enzymes; its aberrant activation enhances antioxidant capacity and limits radiation cytotoxicity (65). In lung cancer, X−ray−activated NRF2 promoted DNA repair via DNA−PKcs and Rad51, with NRF2 knockout increasing MAPK phosphorylation (66). Increased flux through the PPP boosts NADPH production, fueling antioxidant defenses and DNA repair (67, 68). Enhanced mitochondrial biogenesis and metabolic reprogramming redistribute resources for ROS detoxification while sustaining ATP production (26, 69, 70). Cancer cells may shift between OXPHOS and glycolysis to manage oxidative damage. Finally, mitophagy eliminates damaged mitochondria, preserving redox balance (45). Together, these adaptive mechanisms enable cancer cells to withstand radiation−induced oxidative stress, offering targets to overcome radioresistance.

### 2.3 Mitochondrial dynamics: the role of fusion and fission in radioresistance

Mitochondrial dynamics, governed by key GTPases orchestrates fission (dynamin-related protein 1, Drp1) and fusion (mitofusins, Mfn1/2; optic atrophy 1, OPA1), modulate cellular responses to radiation stress (71, 72). In breast cancer, enhanced Drp1−mediated fission promotes fragmentation, lamellipodia formation, migration, and invasion, whereas Mfn1/2 or OPA1 suppress these processes (73). Mfn1/2 are essential for outer mitochondrial membrane fusion while OPA1 is required for inner membrane fusion, without which fusion remains incomplete (74–76). Thus, the finely tuned balance between mitochondrial fission and fusion orchestrated by Drp1, mfn and OPA1, not only governs mitochondrial architecture but also critically regulates cancer cell motility, invasion, and metastatic potential, positioning these dynamics as promising therapeutic targets to limit tumor progression.

Under radiation, tumor cells alter mitochondrial morphology to enhance survival. Drp1 activation increases fission, inhibiting cytochrome c release and intrinsic apoptosis, thereby promoting radioresistance (77, 78). In hepatocellular carcinoma, Drp1 inhibition suppressed fission and mitophagy, leading to dysfunctional mitochondria and increased apoptosis (79). ROS−mediated fission can also activate NF−κB and suppress TP53 to enhance survival (60, 80). However, Drp1 is not essential for cytochrome c release in some cell types, though it influences bioenergetic responses under stress (81). Fission supports mitophagy, removing damaged mitochondria and sustaining bioenergetics in radioresistant tumors (82). X−ray and carbon−ion irradiation induce Drp1 Ser616 phosphorylation via ERK1/2, causing fragmentation; carbon ions produce more extensive fission (83). Epigenetic upregulation of MiD49/51 increases fission, promoting proliferation and reducing apoptosis (84). Inhibiting fission reduces radiation−induced apoptosis (83), and causes G2/M arrest, reducing lung cancer growth (85). Thus, radiation-induced Drp1-mediated mitochondrial fission serves as a critical pro-survival mechanism that inhibits apoptosis, promotes mitophagy and metabolic reprogramming, and drives radioresistance, whereas blocking fission shifts the balance toward mitochondrial elongation, cell cycle arrest, and enhanced radiation sensitivity -underscoring Drp1 as a compelling therapeutic target to overcome radioresistance.

Conversely, promoting fusion *via* OPA1 and Mfn1/2 can enhance radiosensitivity by preserving mitochondrial integrity, efficient OXPHOS, and ATP production (45, 78). Fusion reduces reliance on glycolysis and metabolic plasticity (86), maintains cristae structure for ETC function and apoptotic signaling, while fragmentation elevates ROS and pro−apoptotic pathways (87). Upregulation of OPA1 and Mfn1/2 increases mitochondrial membrane potential and ATP, sensitizing breast cancer cells to radiation (78). Thus, targeting the fission−fusion balance offers a therapeutic strategy to overcome radioresistance.

### 2.4 mtDNA damage and repair mechanisms in radioresistant tumors

mtDNA is highly susceptible to radiation−induced damage due to its proximity to the electron transport chain (a major ROS source) and lack of protective histones (88). Ionizing radiation causes mtDNA strand breaks, base modifications, and deletions, impairing mitochondrial function (33, 89). Radioresistant cancer cells counter this by upregulating mtDNA repair mechanisms, ensuring survival under genotoxic stress (90, 91). Key enzymes of the BER pathway -DNA glycosylases, AP endonuclease (APE1), and DNA polymerase γ (Pol γ) - correct oxidative lesions and single−strand breaks. Glycosylases excise damaged bases, APE1 cleaves abasic sites, and Pol γ fills gaps with proofreading activity (92). Additional mechanisms, including mismatch repair (MMR) and HR−like processes, also maintain mtDNA in resistant tumors (91, 93). Although specific upregulation of mitochondrial-specific DNA repair proteins - OGG1, MYH, or TFAM in irradiated cancer cells remains unconfirmed, these proteins are known to play key roles in mtDNA repair (94, 95). Mitochondrial biogenesis, regulated by PGC−1α, compensates by promoting replication and replacement of damaged mtDNA. PGC−1α activates NRF1/2 and TFAM synthesis, sustaining mitochondrial function (96). Thus, the coordinated upregulation of mtDNA repair pathways particularly BER, alongside PGC−1α−driven mitochondrial biogenesis enables radioresistant cancer cells to preserve mitochondrial genome integrity and function, representing an adaptive vulnerability that can be therapeutically exploited to enhance radiotherapy efficacy.

### 2.5 Mitochondria-mediated apoptotic pathways and their role in radioresistance

Mitochondria regulate the intrinsic apoptotic pathway, a key determinant of cellular fate after radiotherapy (97). This pathway is controlled by Bcl−2 family proteins that mediate mitochondrial outer membrane permeabilization (MOMP), the point of no return in apoptosis (98–100). Pro−apoptotic members (Bax, Bak) form pores to induce MOMP, while anti−apoptotic proteins (Bcl−2, Bcl−xL, Mcl−1) counteract them (89). In response to radiation−induced DNA damage and oxidative stress, BH3−only proteins (Bid, Bim, PUMA, Noxa) activate Bax/Bak or inhibit anti−apoptotic Bcl−2 members, promoting MOMP (101). MOMP triggers cytochrome c release, apoptosome formation (Apaf−1/cytochrome c/ATP), caspase−9 activation, and downstream executioner caspases (caspase−3/−7), culminating in apoptosis (102–105). Other released intermembrane space proteins such as Smac/DIABLO (neutralizes inhibitor of apoptosis proteins, IAPs) and apoptosis-inducing factor, AIF (caspase−independent chromatin condensation) also promote cell death (102). Radioresistant tumors evade apoptosis by dysregulating this intrinsic pathway (106). Overexpression of anti−apoptotic Bcl−2, Bcl−xL, or Mcl−1 inhibits MOMP, preventing cytochrome c release and caspase activation. In HNSCC and synovial sarcoma, high Bcl−xL expression correlated with radioresistance, and Bcl−xL inhibition synergized with radiation; in NSCLC, Mcl−1 inhibition enhanced radiotherapy irrespective of expression levels (107). Bcl−xL and Mcl−1 overexpression also promoted error−prone DNA repair (108). In oral squamous carcinoma, the long isoform Mcl−1L sequestered Bax/Bak, blocking cytochrome c release (109). Conversely, downregulation or mutation of pro−apoptotic Bax/Bak reduces MOMP induction (110). In pancreatic cancer, a decreased Bax/Bcl−2 ratio is found to be associated with radiation resistance (111). Cells lacking both Bax and Bak undergo autophagy instead of apoptosis after radiation, contributing to radioresistance (112). mtDNA−deficient cells resist TRAIL−induced apoptosis due to impaired Bax activation (113). This imbalance between pro− and anti−apoptotic signals enables survival and proliferation under radiation stress, driving tumor recurrence and therapeutic resistance.

**Figure 2:**
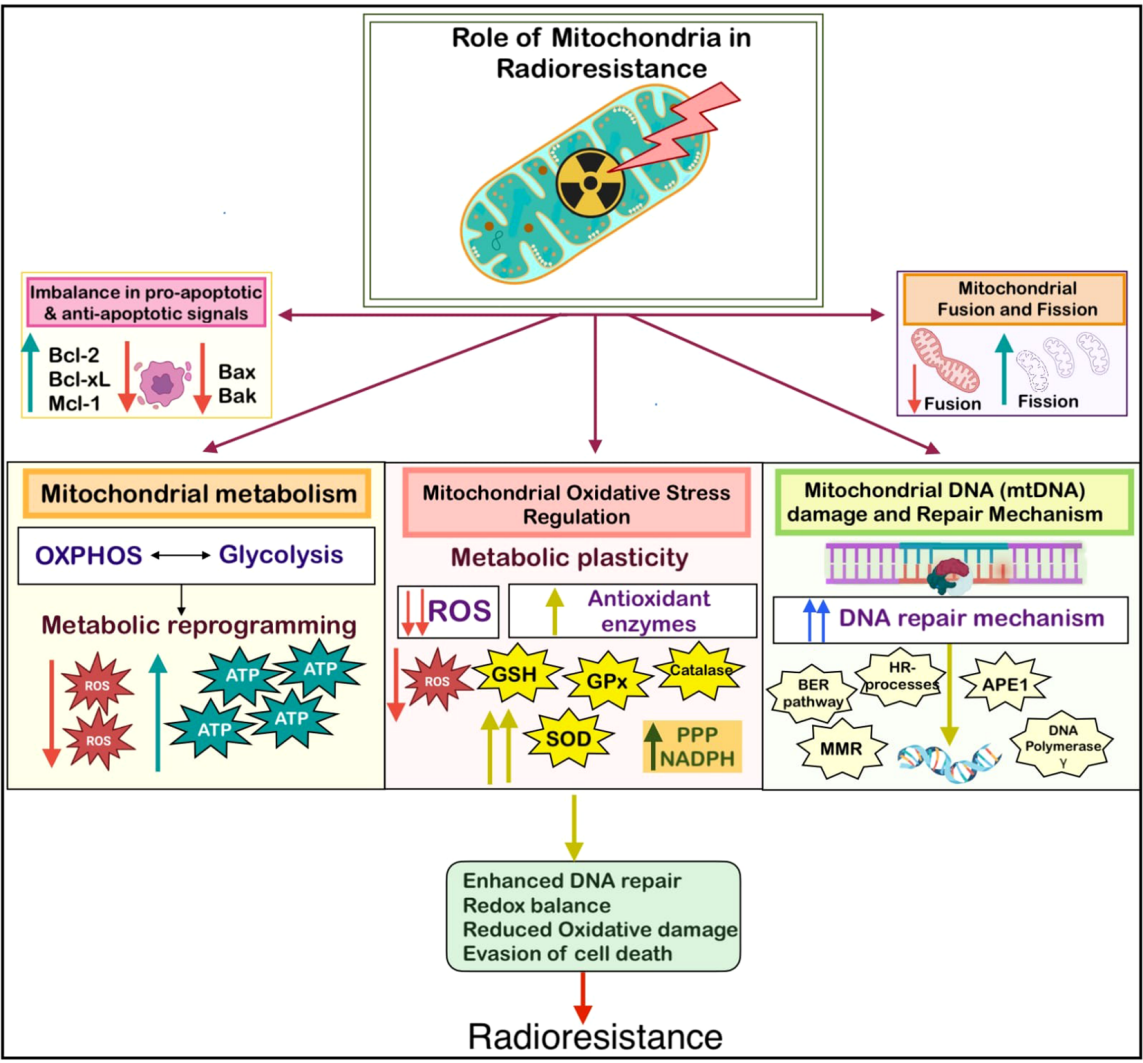
Role of mitochondria in tumor radioresistance: mitochondria plays a central role in promoting radioresistance through multiple interconnected mechanisms. These include an imbalance in pro - and anti-apoptotic signals (upregulated: Bcl-2, Bcl-xL, Mcl-1; downreguled: Bax, Bak), mitochondrial dynamics (fusion and fission), and metabolic reprogramming (OXPHOS and glycolysis shift). In addition, mitochondria regulate oxidative stress via metabolic plasticity, antioxidant enzymes (GSH, GPx, SOD, catalase), and redox balancing pathways (PPP, NADPH). Mitochondrial DNA (mtDNA) repair mechanisms -including BER, HR, and MMR pathways, further enhance survival by reducing oxidative damage and facilitating DNA repair. Collectively, these processes promote enhanced DNA repair, redox homeostasis, oxidative stress reduction, and evasion of cell death, ultimately contributing to tumor radioresistance. OXPHOS, oxidative phosphorylation; ROS, Reactive Oxygen Species; BER, Base Excision Repair; HR, Homologous Recombination; MMR, Mismatch Repair; APE1, Apurinic/apyrimidinic Endonuclease 1.

Collectively, the role of mitochondria in radioresistance highlights their central function as regulators of metabolism, oxidative stress, its dynamics, DNA repair, and apoptosis, together enabling cancer cells to survive radiation-induced stress. Their ability to undergo metabolic reprogramming, maintain redox balance, and evade apoptosis underscores their importance in treatment resistance (**Figure 2**). Thus, targeting mitochondrial bioenergetics, reactive oxygen species (ROS) regulation, and apoptotic pathways therefore represents a promising therapeutic strategy to enhance radiosensitivity and improve radiotherapy outcomes.

## 3 Mitochondrial-targeted therapeutic strategies to overcome radioresistance

### 3.1 Targeting mitochondrial metabolism to enhance radiosensitivity

Pharmacological agents targeting mitochondrial metabolism, such as the OXPHOS inhibitors metformin and phenformin, exhibit radiosensitizing effects in preclinical models (37, 114). Metformin inhibits mitochondrial complex I, reducing ATP production, increasing oxidative stress, and sensitizing cancer cells to radiation (115). It also enhances radiosensitivity of cancer stem cells (CSCs) by decreasing oxygen consumption, mitochondrial membrane potential, and ATP production (116). Shen and colleagues investigated whether modulation of tumor bioenergetic pathways could improve the radiosensitivity of diffuse intrinsic pontine gliomas (DIPGs), which are highly resistant to conventional therapy. The study showed that DIPG cells depend heavily on mitochondrial oxidative phosphorylation (OXPHOS) for energy production and survival following radiation exposure. Pharmacological inhibition of mitochondrial metabolism using OXPHOS-targeting agents such as metformin and phenformin disrupted ATP production, increased oxidative stress, and enhanced radiation-induced tumor cell death. These agents also reduced the capacity of DIPG cells to recover after irradiation, thereby potentiating the cytotoxic effects of radiotherapy. The findings emphasized the role of metabolic reprogramming in DIPG radioresistance and suggested that combining radiotherapy with mitochondrial metabolic inhibitors like metformin and phenformin may represent a promising therapeutic strategy for improving outcomes in this aggressive pediatric brain tumor (117). In hepatocellular carcinoma, phenformin is seen to induce mitochondrial dysfunction and fragmentation, shifting cells to glycolysis (118). The mitochondria−targeted metformin analog Mito−Met_10_ more potently inhibits complex I, stimulates superoxide generation, and activates AMPK, enhancing radiosensitivity (119). Additionally, glycolysis inhibitor 2−deoxyglucose (2−DG) exacerbates oxidative stress and promotes apoptosis in breast cancer cells *in vitro* (120). In colorectal cancer cells, 2−DG induces G0/G1 arrest and mitochondrial apoptosis *via* p21 and p53, independent of ATP levels (121). Using human cancer cell lines with different p53 statuses, Sinthupibulyakit and co-workers found its radiosensitizing effect is p53−dependent (122). Combining ETC inhibitors with 2−DG increases ROS and cytotoxicity as observed in human colon carcinoma cells (123).

- Beyond traditional metabolic inhibitors, novel compounds targeting specific mitochondrial respiratory complexes are emerging as radiosensitizers. Atpenin A5 (complex II inhibitor) impairs ATP production and elevates ROS (124), while antimycin A (complex III inhibitor) enhances oxidative stress and cytotoxicity (125); notably, it also suppresses cancer stem cell stemness and synergizes with gefitinib in resistant lung cancer models (126). Cyanide derivatives (complex IV inhibitors) and ATP synthase inhibitors like oligomycin similarly disrupt oxidative phosphorylation, increase ROS, and promote radiosensitization in a concentration-dependent manner (127–129). Collectively, these agents demonstrate that selective mitochondrial respiratory chain disruption whether at electron transfer complexes or ATP synthesis consistently converges on ROS-mediated mechanisms to potentiate radiation-induced tumor cell death, positioning mitochondrial metabolism as a critical target for enhancing radiotherapy efficacy.
- Another promising strategy involves targeting the metabolic dependencies of cancer cells by inhibiting key metabolic enzymes. The glutaminase inhibitor CB−839 (telaglenastat) depletes glutamate, impairs the TCA cycle and mitochondrial respiration, increases ROS, and enhances radiation−induced DNA damage in head and neck squamous cell carcinoma, lung tumors, and chronic lymphocytic leukemia (130–132). Combining metabolic inhibitors with immunotherapy or DDR inhibitors may further improve radiotherapy efficacy. The OXPHOS inhibitor IACS−010759 enhanced anti−PD−1 therapy and radiotherapy in NSCLC by reducing regulatory T cells and increasing CD8^+^ T cell activity (133). The ATR inhibitor ceralasertib (AZD6738) blocks radiation−induced PD−L1 upregulation, potentiating T cell−mediated antitumor responses (134).

### 3.2 Modulating mitochondrial ROS to improve radiation efficacy

**Figure 3:**
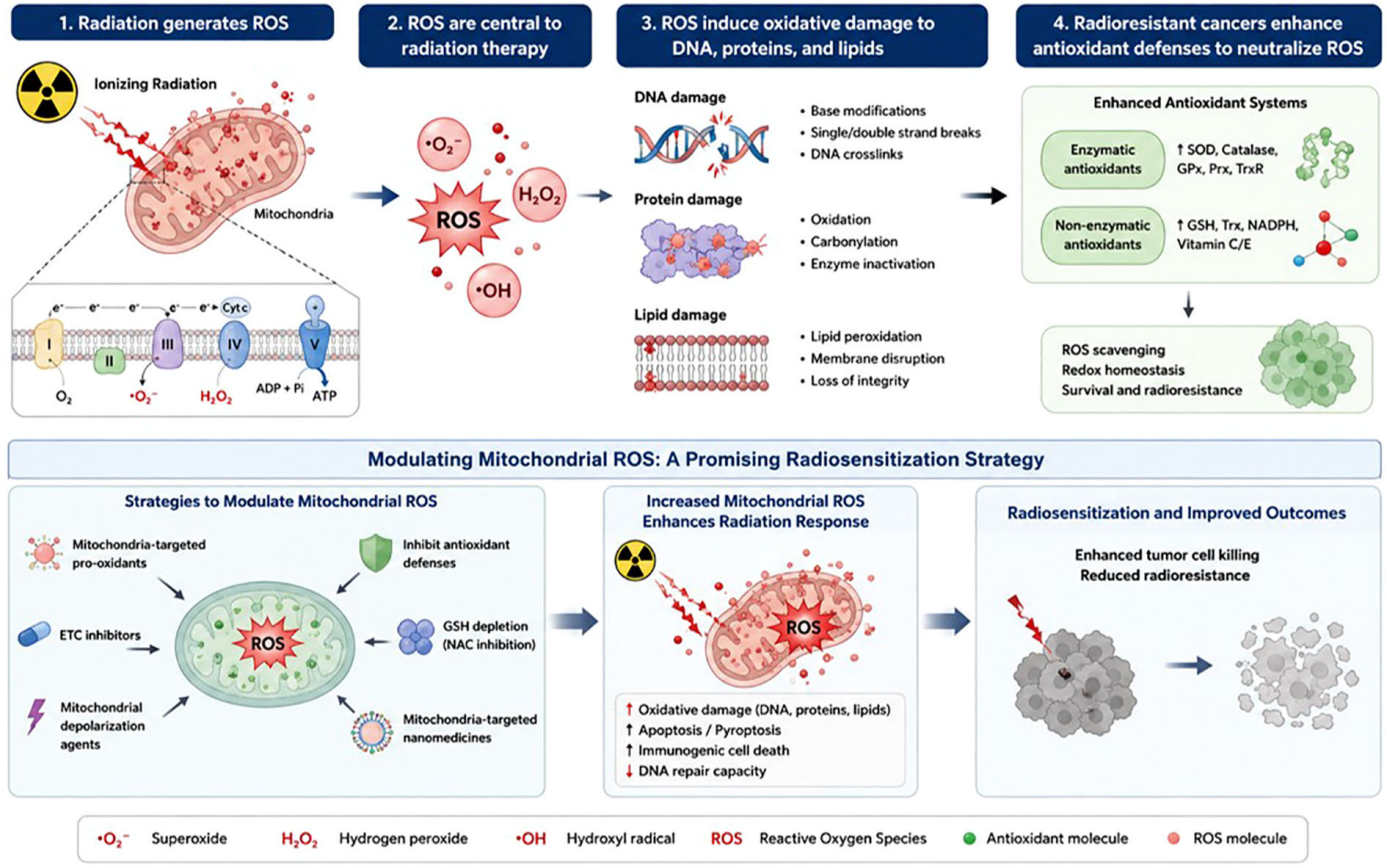
Mitochondrial ROS in radiation therapy and radiosensitization. Ionizing radiation induces mitochondrial electron leakage from the electron transport chain (ETC), resulting in the generation of reactive oxygen species (ROS), including superoxide anion (•O_2_^-^), hydrogen peroxide (H_2_O_2_), and hydroxyl radicals (•OH). These ROS act as central mediators of radiation-induced cytotoxicity by causing oxidative damage to DNA, proteins, and membrane lipids, leading to genomic instability, mitochondrial dysfunction, and cell death. Radioresistant tumor cells counteract ROS accumulation through upregulation of enzymatic antioxidants, such as superoxide dismutase (SOD), catalase, glutathione peroxidase (GPx), peroxiredoxins (Prx), and thioredoxin reductase (TrxR), as well as non-enzymatic antioxidant systems including glutathione (GSH), thioredoxin (Trx), NADPH, and vitamins C/E, thereby maintaining redox homeostasis and promoting survival. The lower panel illustrates therapeutic strategies to modulate mitochondrial ROS, including mitochondria-targeted pro-oxidants, ETC inhibitors, mitochondrial depolarization agents, antioxidant defense inhibitors, GSH depletion, and mitochondria-targeted nanomedicines. Elevation of mitochondrial ROS enhances oxidative stress, apoptosis/pyroptosis, immunogenic cell death, and impairment of DNA repair pathways, ultimately improving radiosensitization and therapeutic efficacy while reducing tumor radioresistance.

ROS are central to radiation therapy, inducing oxidative damage to DNA, proteins, and lipids (55, 135). Radioresistant cancers enhance antioxidant defenses to neutralize ROS (136); thus, modulating mitochondrial ROS levels is a promising radiosensitization strategy [**Figure 3]**. Inhibiting glutathione (GSH) metabolism depletes the major intracellular antioxidant, increasing radiation sensitivity (137). Buthionine sulfoximine (BSO) inhibits γ−glutamylcysteine synthetase, the rate−limiting enzyme in GSH synthesis (138). BSO disrupts redox balance, enhances oxidative stress, and sensitizes tumors to radiation (138) and estradiol−induced apoptosis (139), while inhibiting pancreatic cancer proliferation (140). A phase I trial (NCT00005835) is evaluating BSO plus melphalan in pediatric neuroblastoma. Mitochondrial ROS inducers such as elesclomol (a copper ionophore) disrupt ETC function, elevating ROS and selectively killing tumor cells (141, 142). Elesclomol impairs mitochondria in glioblastoma stem−like cells (143), and induces copper−dependent oxidative stress in GNAQ/11−mutant uveal melanoma (144). Radiotherapy induces cuproptosis via CTR1 upregulation and GSH depletion, and combining RT with cuproptosis inducers overcomes radioresistance (145). Menadione (146) and β−lapachone (147) also enhance radiation responses through ROS generation.

Targeting mitochondrial SOD can also radiosensitize. Reducing mitochondrial superoxide attenuates Akt/ERK survival signaling (148), while ATN−224 (SOD1 inhibitor) induces apoptosis in malignant hematopoietic cells with therapeutic selectivity (149). Combinatorial approaches include NQO1−bioactivated β−lapachone (ARQ761), which amplifies ROS and DNA damage in NQO1−positive NSCLC and HNSCC, causing synergistic radiosensitization (150, 151). A phase I trial showed manageable toxicity and early antitumor activity (152). PARP inhibitors (olaparib, veliparib) also increase ROS and enhance radiosensitivity in TP53−mutant cancers (153), endometrial carcinoma (154), and triple−negative breast cancer (phase I trial) (155). However, careful optimization of dosing and sequencing is needed to minimize toxicity (156).

Nanoparticle−based delivery systems enable targeted ROS modulation. Arsenic trioxide (ATO) loaded on nano−zirconia achieves mitochondrial targeting with reduced systemic toxicity (157). Wang et al. developed a mitochondria-responsive arsenic(III)-based nanomicelle platform designed to induce pyroptosis and enhance synergistic cancer immunotherapy. The study demonstrated that the engineered nanomicelles efficiently delivered arsenic(III) to tumor cells, where they triggered mitochondrial dysfunction, excessive ROS generation, and activation of inflammasome-mediated pyroptotic cell death pathways characterized by gasdermin cleavage and inflammatory cytokine release. Unlike conventional apoptosis, pyroptosis promoted strong immunogenic cell death, leading to enhanced dendritic cell maturation, T-cell activation, and remodeling of the immunosuppressive tumor microenvironment. The nanoplatform also showed synergistic antitumor efficacy when combined with immunotherapeutic strategies by amplifying systemic antitumor immune responses and inhibiting tumor growth and metastasis in experimental models (158). Cerium oxide nanoparticles (CeO_2_ NPs) induce cytotoxicity via ROS, GSH depletion, and mitochondrial damage in melanoma, colorectal, and lung cancer cells while sparing normal cells (159–161). ROS−activatable nanoprodrugs (e.g., HTCF) trigger Fenton reactions and amplify oxidative stress (162). Mitochondria−targeted blended nanoparticles enable synergistic chemo−photodynamic therapy (163). Copper−artemisinin nanoprodrugs (Cu−ART NPs) catalyze ROS generation, disrupting mitochondrial membrane potential and triggering apoptosis/ferroptosis (164). Dual−targeted mesoporous silica−liposome nanoassemblies loaded with paclitaxel achieve eight−fold higher efficacy in NSCLC (165). Despite strong preclinical evidence, clinical translation of nanoparticle−based ROS modulators remains unrealized; challenges include normal tissue toxicity, addressed by tumor−selective designs and nanocarriers (liposomes, polymeric, inorganic) that minimize systemic effects (166, 167).

Biomarkers of tumor ROS sensitivity can guide patient stratification. A ROS−based scoring system in ovarian cancer correlates with cisplatin sensitivity and prognosis (168). An oxidative stress–related model stratifies bladder cancer risk and treatment response (169). A five−gene mitochondrial/oxidative stress signature (CTSL, TXNRD2, NUDT1, STOX1, CYP2E1) predicts glioblastoma survival; silencing NUDT1 increases ROS and apoptosis (170). Machine learning−derived ROS risk models stratify TNBC patients by survival and immune landscape (166). A ROS related gene signature also stratifies hepatocellular carcinoma outcomes and drug sensitivity (171). Together, these findings establish that pharmacological targeting of mitochondrial metabolism, whether through established OXPHOS and glycolysis inhibitors or next-generation respiratory complex disruptors converges on ROS mediated mechanisms to potentiate radiation-induced tumor cell death, while combinatorial strategies integrating metabolic modulation with immunotherapy or DDR inhibition offer a promising translational avenue to overcome radioresistance and improve clinical outcomes.

### 3.3 Targeting mitochondrial dynamics to induce radiosensitivity

Drp1 mediates mitochondrial fission. Its overactivation promotes excessive fragmentation, which is associated with apoptosis resistance and enhanced cancer cell survival after radiotherapy (172, 173). Inhibiting Drp1 shifts the balance toward fusion, promoting apoptosis and increasing radiosensitivity. Yamamori et al. showed that Drp1 inhibition during X−irradiation impairs fission, leading to mitochondrial elongation, DNA damage accumulation, and mitotic catastrophe (174). Zhang et al. found that cytoplasmic irradiation induces ROS and loss of membrane potential, activating Drp1−dependent fission; Drp1 inhibition reduced fragmentation and improved survival (175). The small molecule Mdivi−1 inhibits Drp1 GTPase activity, reducing fission and sensitizing cancer cells to radiation by promoting apoptosis, though it may also affect complex I and ROS production (176). Sodium butyrate (NaBt) downregulates DRP1 and its activating Ser616 phosphorylation via cyclin B1–CDK1 suppression, promoting fusion and apoptosis in colorectal cancer cells (177). Mdivi−1 synergizes with platinum therapy by disrupting fission, enhancing MOMP, ROS, and cytochrome c release, without affecting normal cells (178). Invasive breast cancer cells upregulate Drp1−mediated fission and downregulate fusion proteins (MFN1/2, OPA1); Drp1 inhibition reduces fragmentation, lamellipodia formation, migration, and metastasis in xenografts (73). More selective Drp1 inhibitors, such as Drpitor1 and Drpitor1a, are under development which selectively inhibit Drp1’s GTPase function with higher fidelity. These novel agents may offer better therapeutic indices suitable for human testing (173, 179). Thus, targeting Drp1-mediated mitochondrial fission represents a promising radiosensitization strategy that not only restores apoptotic susceptibility and mitotic fidelity but may also curtail metastatic dissemination, with the emergence of more selective inhibitors poised to refine therapeutic specificity.

Enhancing mitochondrial fusion is another approach to counteract radioresistance. Autophagy inhibition preserves mitochondrial integrity by upregulating OPA1 and MFN2 while downregulating DRP1, reducing neuronal injury after irradiation (180). In pancreatic cancer, DRP1 inhibition or MFN2 overexpression enhances mitochondrial connectivity, reduces ATP production, activates mitophagy, and sensitizes cells to apoptosis, suppressing tumor growth (181). Conversely, OPA1 is upregulated in triple−negative breast cancer (TNBC) and associated with poor prognosis; OPA1 inhibition reduces proliferation, migration, and invasion via tumor−suppressive miRNAs (148/152 family) without impairing respiration, identifying OPA1 as a druggable target (182). Although direct clinical evidence linking enhanced fusion to reduced radioresistance remains limited, these mechanistic insights provide a foundation for developing selective modulators of mitochondrial dynamics. Ongoing research should aim to identify more selective and potent modulators of mitochondrial dynamics, as well as to elucidate the precise molecular mechanisms by which these alterations influence radiosensitivity.

### 3.4 Targeting mtDNA repair pathways

Targeting mtDNA repair pathways represents a promising strategy to overcome radioresistance. Inhibition of Pol γ, the principal enzyme for mtDNA replication and repair, disrupts genome integrity, leading to oxidative damage accumulation, respiratory failure, and heightened vulnerability to radiation-induced stress (183, 184). Sasaki et al. showed that Pol γ suppression impairs mtDNA synthesis, dissipates mitochondrial membrane potential, elevates ROS, and triggers cytochrome c–mediated caspase activation, with preferential toxicity toward malignant cells (185). Somuncu et al. further identified the small-molecule Pol γ inhibitor congo red (CR), which blocks strand displacement and DNA synthesis. In MLH1-deficient colon cancer, CR induces mitochondrial dysfunction, oxidative mtDNA lesions, and ROS accumulation, suppressing tumor growth *in vitro* and *in vivo* while sparing MLH1-proficient cells, thus exemplifying a synthetic lethal strategy (184). Other mtDNA maintenance proteins also govern radiosensitivity. SSBP1 downregulation destabilizes mtDNA replication, reducing ATP production, elevating ROS, and amplifying radiation-induced DNA damage and apoptosis in non-small cell lung cancer (186). Likewise, EXOG, a mitochondrial 5′-exonuclease/endonuclease essential for single-strand break repair (mtSSBR), prevents accumulation of unrepaired breaks; its loss causes mitochondrial dysfunction, membrane potential collapse, ROS burst, and intrinsic apoptosis, establishing EXOG as a critical target for radiosensitization (187). Mitochondrial-targeted nucleoside analogs provide a complementary approach by incorporating into mtDNA to induce chain termination and genomic instability. Alovudine, a thymidine dideoxynucleoside and Pol γ inhibitor, selectively depletes OXPHOS capacity and ATP in acute myeloid leukemia, promoting differentiation and reducing leukemic burden in xenografts (188). Zalcitabine (ddC) enhances radiation sensitivity across diverse cancer lines by disrupting mtDNA replication and mitochondrial function (189), whereas fialuridine (FIAU) inhibits Pol γ processivity, reducing mtDNA copy number and inducing structural defects that compromise organelle integrity (190). Intriguingly, Wang et al. demonstrated that combining liposomal doxorubicin with radiotherapy and anti–PD-1 enhances abscopal responses in a mtDNA integrity–dependent manner *via* cGAS/STING activation and type I interferon signaling, linking mtDNA damage to systemic antitumor immunity (191).

Collectively, these findings establish that disruption of mtDNA replication and repair -whether through Pol γ inhibition, destabilization of ssDNA-binding or break-repair machinery, or nucleoside analog-mediated chain termination - converges on mitochondrial dysfunction, oxidative stress amplification, and apoptotic priming to surmount radioresistance. Optimizing the specificity and therapeutic index of these strategies remains essential for clinical translation in radiation-refractory malignancies.

### 3.5 Targeting mitochondrial apoptotic pathways for enhanced radiosensitization

Targeting mitochondrial apoptotic pathways is a promising radiosensitization strategy (192). BH3 mimetics are small molecules that antagonize anti−apoptotic Bcl−2 family members, restoring apoptosis in resistant tumors (193). ABT−737 and its oral derivative venetoclax (ABT−199) selectively inhibit Bcl−2, disrupting its interaction with Bax/Bak, inducing MOMP, cytochrome c release, and caspase activation (194, 195). ABT−737 enhances radiation−induced apoptosis in cervical cancer via caspase activation and mitochondrial dysfunction (196), and in radioresistant breast cancer by promoting Bcl−2/Bcl−xL inhibition (197). Venetoclax synergizes with radiation in diffuse midline glioma, reducing viability and tumor growth through caspase−mediated apoptosis (198), and lowers the apoptotic threshold in malignant B cells, potentiating radioimmunotherapy (199).

Bcl−xL inhibitors (A−1331852) enhance radiation−induced PARP/caspase−3 cleavage and delay tumor growth in xenografts; PTEN loss or Mcl−1 overexpression abrogates this effect (200). In mesothelioma, A−1331852 exhibits cytotoxicity (EC_50_ 0.13–1.42 μM) and radiosensitization (enhancement ratio 1.3–1.8) (201). In HNSCC models, A−1155463, A−1331852, and navitoclax synergize with irradiation, reducing clonogenic survival; A−1331852 additionally suppresses invasion (202). The Mcl−1 inhibitor S63845 combined with radiation reduces viability and colony formation in radioresistant oral squamous cell carcinoma (OSCC) lines and delays xenograft tumor growth with increased cleaved caspase−3/PARP (203).

Resistance also arises from inhibitor of apoptosis proteins (IAPs) (XIAP, c−IAP1/2, survivin) and persistent NF−κB signaling (204–206). Smac mimetics (LCL161, birinapant) antagonize IAPs, promote cIAP degradation, enhance caspase−dependent apoptosis, and radiosensitize head−and−neck cancer, glioblastoma, and other models (207–209). Birinapant is being tested in a phase I trial with re−irradiation (NCT03803774); LCL161 has progressed through phase I/II studies, though mostly with chemotherapy rather than radiotherapy (210, 211).

**Figure 4:**
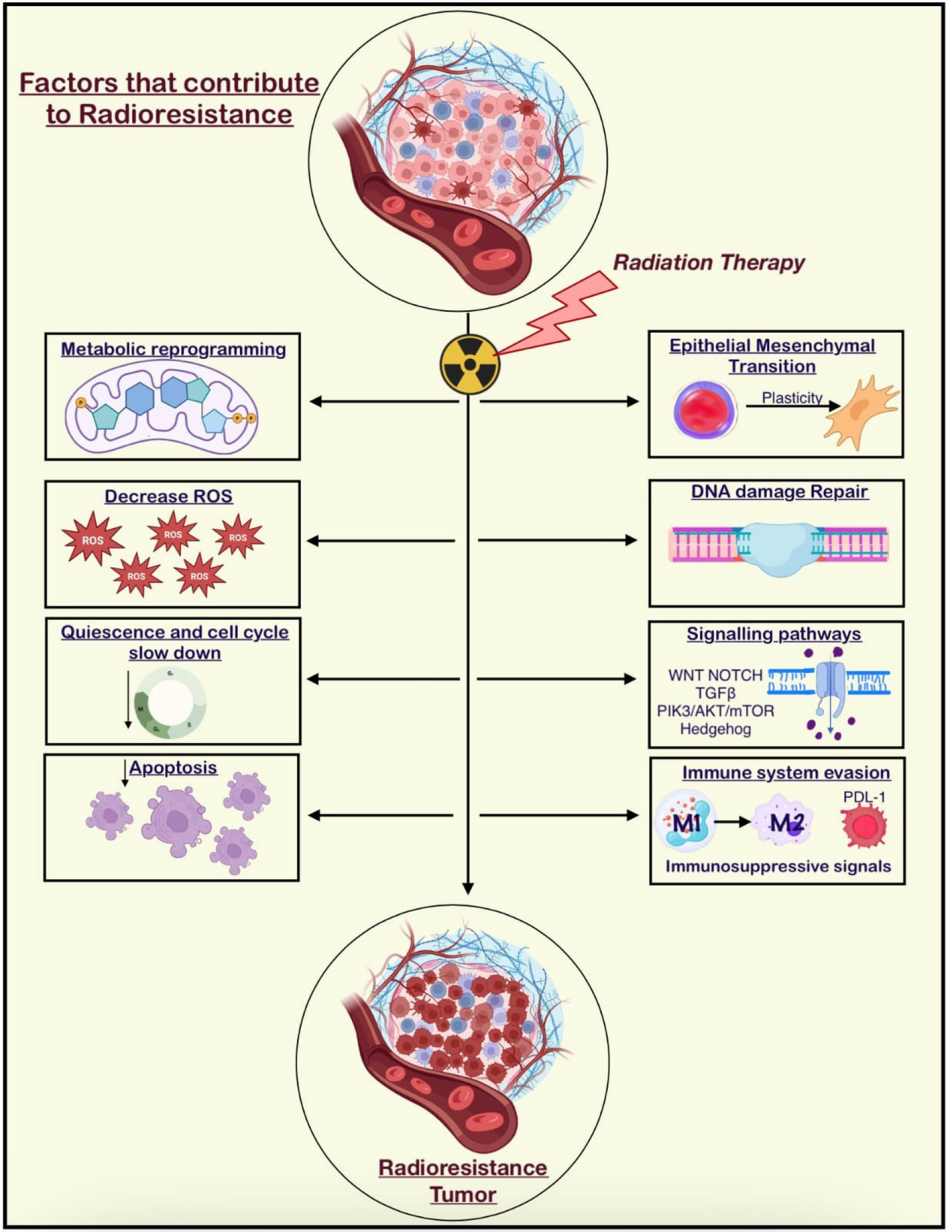
Factors contributing to tumor radioresistance and therapeutic targeting: schematic illustration shows the key cellular and molecular mechanisms that enable tumor cells to resist radiation therapy, that could be potential therapeutic targets. These include metabolic reprogramming (to sustain energy production), oxidative stress regulation and reduction of reactive oxygen species (ROS), quiescence state and slower cell cycle, and apoptosis evasion. Additional contributors are epithelial–mesenchymal transition (EMT), mitochondrial DNA (mtDNA) damage and enhanced DNA damage repair, activation of pro-survival signaling pathways (WNT, NOTCH, TGFβ, PI3K/AKT/mTOR, Hedgehog), and immune system evasion through immunosuppressive signals. Together, these processes drive the development of a radioresistant tumor phenotype, limiting therapeutic efficacy.

In summary, novel therapeutic strategies targeting mitochondrial metabolism, ROS modulation, and apoptosis pathways have demonstrated promising radiosensitizing effects in preclinical models and may overcome the limitations of current radiotherapy approaches [**Figure 4]**. Continued research into mitochondrial-targeted interventions has the potential to improve clinical outcomes for patients with treatment-resistant tumors.

Further, Wang and colleagues reviewed recent advances in mitochondria-targeted molecular tools for precise tumor therapy, highlighting mitochondria as central regulators of cancer metabolism, ROS production, apoptosis, and therapeutic resistance. The authors discussed multiple targeting platforms, including small molecules, peptides, nanoparticles, AIEgens, and metal-based systems functionalized with mitochondrial-targeting ligands such as triphenylphosphonium (TPP) and mitochondria-penetrating peptides to achieve selective mitochondrial accumulation in tumor cells. These strategies induce mitochondrial dysfunction through ROS amplification, ATP depletion, membrane depolarization, and cytochrome-c-mediated apoptosis, thereby enhancing tumor cell killing. The review also emphasized the integration of mitochondrial targeting with advanced modalities such as photodynamic, photothermal, chemodynamic, and sonodynamic therapies, as well as immunotherapy and gene therapy, to improve treatment precision and overcome therapy resistance. Furthermore, the authors highlighted the promise of stimulus-responsive nanoplatforms capable of controlled drug release within the tumor microenvironment while acknowledging ongoing challenges related to biosafety, delivery efficiency, off-target toxicity, and clinical translation (212).

## 4 Conclusion

Mitochondria orchestrate radioresistance through interconnected mechanisms: metabolic reprogramming (enhanced OXPHOS, glycolysis, and PPP flux), ROS regulation (upregulated antioxidant enzymes, GSH, NRF2), dynamic remodeling (Drp1−mediated fission, OPA1/Mfn fusion), apoptotic dysregulation (Bcl−2 family overexpression, IAP−mediated caspase blockade), and mtDNA repair (Pol γ, SSBP1, EXOG). These adaptations collectively enable cancer cells to survive radiation−induced genotoxic and oxidative stress, repair damage, and evade cell death.

Targeting these mitochondrial vulnerabilities offers a powerful strategy to radiosensitize resistant tumors. Pharmacological agents have demonstrated preclinical efficacy: OXPHOS inhibitors (metformin, phenformin, IACS−010759), ROS modulators (BSO, elesclomol, β−lapachone), fission inhibitors (Mdivi−1, Drpitor1), pro−apoptotic BH3 mimetics (venetoclax, A−1331852, S63845), Smac mimetics (birinapant, LCL161), and mtDNA repair disruptors (Pol γ inhibitors, nucleoside analogs). Combinatorial approaches with immune checkpoint inhibitors (e.g., IACS−010759 + anti−PD−1) and nanoparticle−based delivery systems further amplify efficacy while minimizing toxicity.

To translate these insights into clinical practice, future research must: (i) identify predictive biomarkers of mitochondrial vulnerability (e.g., ROS signatures, Drp1 expression, Bcl−2 family profiles) to stratify patients; (ii) optimize drug−radiation sequencing and dosing to maximize synergy and limit normal tissue toxicity; and (iii) develop selective, bioavailable mitochondrial modulators suitable for human trials. A deeper mechanistic understanding of mitochondrial radioresistance will enable personalized radiotherapy regimens that exploit cancer−specific metabolic and apoptotic dependencies, ultimately improving outcomes for patients with treatment−resistant malignancies.

## 5 Future perspective

Emerging evidence positions mitochondria as actionable hubs for radiosensitization, yet several hurdles remain. First, predictive biomarkers—such as ROS responsiveness, Drp1 phosphorylation status, or Bcl−2 family expression profiles—must be validated to stratify patients most likely to benefit. Second, next−generation mitochondrial modulators (e.g., Drpitor1a, Mito−Met_10_) require rigorous pharmacokinetic and safety evaluation, ideally within tumor−selective nanocarriers to spare normal tissues. Third, rational combination regimens should exploit synthetic lethal interactions (e.g., Pol γ inhibition in MLH1−deficient tumors) and synergies with immunotherapy (cGAS/STING activation). Fourth, real−time monitoring of mitochondrial dynamics and metabolic flux using advanced imaging or liquid biopsies could guide adaptive radiotherapy. Finally, clinical trials must address optimal sequencing, dosing, and fractionation when pairing mitochondrial agents with radiation. Conquering radioresistance will likely demand personalized, mitochondria−targeted adjuncts that convert refractory tumors into radiosensitive phenotypes.
